# Ischemia with No Obstructive Coronary Artery Disease (INOCA): A Review

**DOI:** 10.3390/life15101554

**Published:** 2025-10-03

**Authors:** Laura Viola, Megan Masters, Umar Shafiq, Krishnam Raju Jujjavarapu, Suvitesh Luthra

**Affiliations:** 1 Division of Cardiac Surgery, University Hospital Southampton, Southampton SO16 6YD, UK; 2 Human Development and Health, Faculty of Medicine, University of Southampton, Southampton SO17 1BJ, UK

**Keywords:** INOCA, ischemia, coronary artery disease, surgical treatment, non-obstructive coronary artery disease, myocardial ischemia

## Abstract

Background: Ischemia with no obstructive coronary artery disease (INOCA) is characterized by myocardial ischemia in the absence of significant coronary artery stenosis. Despite the lack of major obstructive lesions, patients often present with chest pain, making diagnosis and management a significant challenge. Materials and Methods: A comprehensive search strategy of electronic databases (2000 to 2024) was used to identify studies assessing pathophysiology, diagnosis, surgical treatments, interventions, and outcomes in INOCA. Clinical trials, observational studies, case-control studies, and cohort studies were included. Results: Emerging surgical treatments may have a role in certain subgroups of INOCA patients, particularly those with severe and persistent symptoms or underlying pathophysiological factors that do not respond adequately to pharmacological therapies. Transmyocardial revascularization (TMR) and sympathetic denervation procedures reduce coronary vasospasm in refractory angina. Trials have shown promise for coronary sinus occlusion. Autologous stem cell therapy is an innovative surgical approach that has shown promise in early trials but remains investigational. Selective surgical cardiac vein retroperfusion remains largely experimental, with limited clinical data. Conclusions: This review highlights the need for ongoing research and clinical trials to assess the effectiveness of surgical and nonsurgical options in INOCA. Although current data on surgical interventions is limited, these treatments may offer hope for patients with refractory symptoms. A personalized and multidisciplinary approach to management is essential for optimal patient outcomes.

## 1. Introduction

Ischemia with no obstructive coronary artery disease (INOCA) refers to a clinical condition characterized by myocardial ischemia in the absence of significant coronary artery stenosis. INOCA represents a challenge, as patients frequently present with typical ischemic symptoms such as chest pain and dyspnoea, but conventional diagnostic tests do not reveal significant lesions in the coronary arteries. These can be caused by underlying pathophysiological mechanisms, such as microvascular dysfunction, coronary vasospasm, or endothelial dysfunction, with reduced myocardial blood supply resulting in acute and chronic symptoms of ischemia [1].

The ACC National Cardiovascular Data Registry estimates that 3–4 million people have signs and symptoms of INOCA annually, with a striking female predominance [2]. Estimates are largely based on negative angiograms in patients with angina. Up to 50% of females and 7–10% of males with typical symptoms lack angiographic evidence of epicardial coronary artery disease and may have INOCA [2,3]. While traditionally overshadowed by obstructive coronary artery disease (CAD), recognition of INOCA has been increasing with advancements in diagnostic imaging techniques for coronary microcirculation and myocardial perfusion. Patients with INOCA may remain undiagnosed, leading to significant workdays lost, increased healthcare costs from further investigations, and repeated clinic/hospital attendance for unresolved symptoms. However, managing INOCA remains difficult: conventional treatments such as pharmacological therapy, lifestyle modifications, and medical management alone often fail to provide adequate relief for many patients. Emerging percutaneous nonpharmacological strategies are being explored in clinical practice and research [4].

This systematic review aims to critically evaluate the available evidence on nonpharmacological treatment options for INOCA and provide an overview of the current role of surgery in managing this complex condition. This may help refine surgical indications and improve patient outcomes in the management of INOCA.

### 1.1. Pathophysiology of INOCA

**Figure 1:**
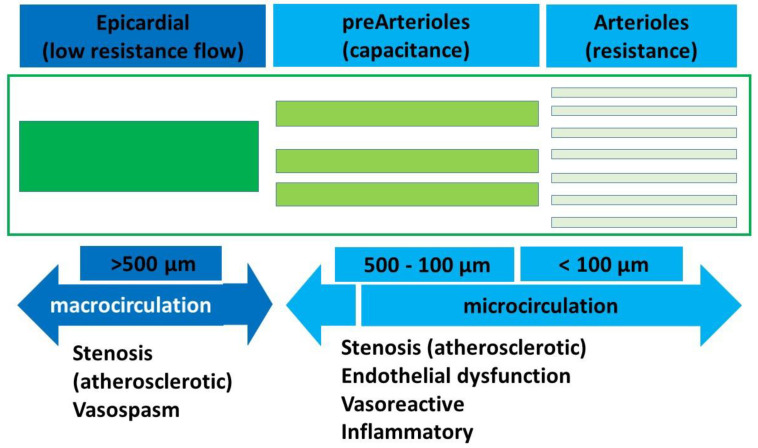
Pathophysiology of micro- and macrocirculation.

Microvasculature typically involves the pre-arteriolar vessels (<500 µm) and arterioles (<100 µm). The pre-arteriolar vessels are conductance vessels, and the arteriolar vessels are the resistance vessels in the coronary circulation. The pathophysiology and endotypes of INOCA can be largely divided into the putative mechanisms affecting these two groups of microvessels as obstruction, vasospasm, or a combination of the two (Figure 1,Table 1). Microvascular dysfunction, coronary vasospasm, endothelial dysfunction, and inflammation are the main recognized underlying causes of INOCA. Other less understood mechanisms include subclinical atherosclerosis, hormonal, autonomic, genetic, and environmental factors. The pathophysiology of INOCA is multifactorial; understanding these underlying mechanisms is essential for improving diagnostic accuracy and developing effective therapeutic strategies [5,6].

### 1.2. Diagnostic Approach to INOCA

The diagnosis of INOCA is challenging due to the lack of visible obstruction on traditional tests. Advanced diagnostic strategies are needed to assess the functionality of the coronary microcirculation, as well as to detect other possible causes of ischemia such as endothelial dysfunction or vasospasm [7,8]. There is a lack of standardization of the terminology and putative mechanisms, which limits meaningful comparison of different cohorts and treatments.

**Figure 2:**
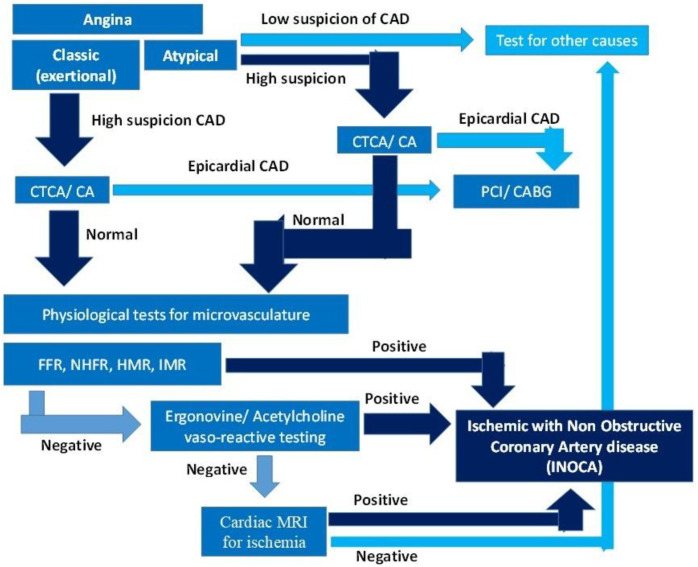
Diagnostic algorithm for INOCA.

The diagnostic tools and approaches currently used to diagnose INOCA include imaging modalities and physiological tests of flow and resistance in the microcirculation, which are the mainstay for diagnosis. A diagnostic algorithm is provided in Figure 2, and the list of various tests that are used is shown in Table 2.

## 2. Material and Methods

**Figure 3:**
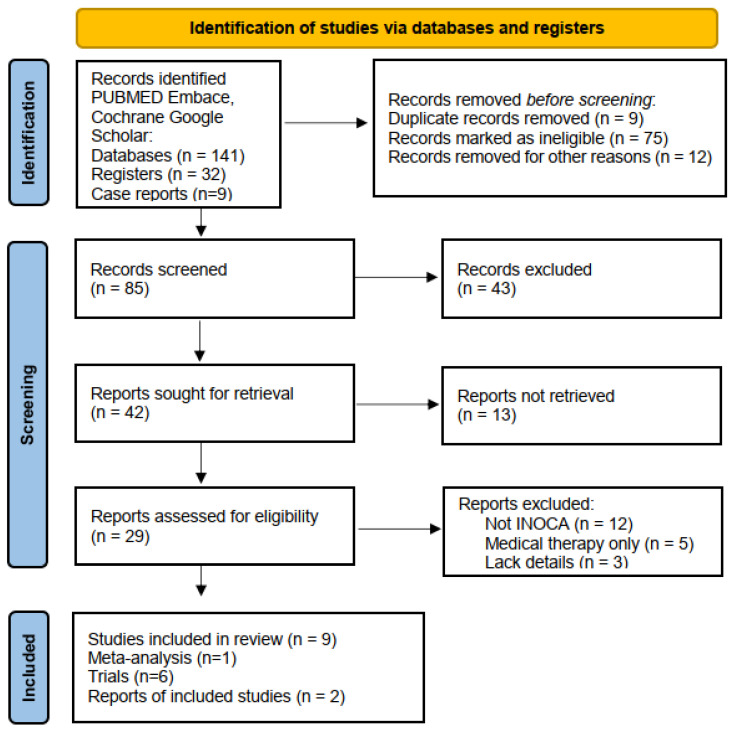
PRISMA (Preferred reporting items for systematic reviews and meta-analysis) diagram for selection of studies.

A thorough search strategy was developed to locate all relevant studies. The review adhered to the PRISMA guidelines, ensuring transparency and replicability in methodology. The following electronic databases were explored for articles published between 2015 and 2024: PubMed, Embase, Cochrane Library, and Google Scholar. The search terms included various combinations of keywords such as “Ischemia with No Obstructive Coronary Artery Disease,” “INOCA,” “Microvascular Dysfunction,” “Coronary Artery Bypass Grafting,” “Percutaneous Coronary Intervention,” “Transmyocardial Revascularization,” “Stem Cell Therapy,” “Sympathectomy,” “Coronary sinus reducer”, and “Surgical Treatment in INOCA.” We aimed to identify studies that examined surgical treatment interventions, outcomes, and pathophysiology in INOCA. No limitations were applied regarding the study type, but only studies meeting the inclusion criteria were selected for further analysis. Inclusion criteria encompassed studies published in peer-reviewed journals that focused on surgical procedures or interventions for INOCA patients (ESC 2023—PRISMA diagram Figure 3). A narrative synthesis was performed from the included studies due to heterogeneity in study designs and outcomes.

### 2.1. Conventional Non-Surgical Treatment of INOCA

Medical therapy is the mainstay in the treatment of INOCA and aims to reduce symptoms and improve quality of life.

#### 2.1.1. Pharmacologic Therapy

Pharmacological management of INOCA focuses to improve endothelial function, optimize coronary blood flow, and manage ischemic symptoms (Table 3). The treatment aims to increase myocardial blood supply by reducing spasm, promoting vasodilation, and to reduce myocardial oxygen demand by reducing heart rate/contractility. Preventative therapies aim to reduce the progression of the atherosclerotic burden of obstructive disease.

#### 2.1.2. Lifestyle Modifications

Lifestyle changes help reduce the overall burden on the cardiovascular system and improve coronary microcirculation.

### 2.2. Nonpharmacological Treatment Options for INOCA

Surgical treatment options can be considered in specific circumstances, in which pharmacological therapy cannot control the symptoms or when microvascular dysfunction is accompanied by other coronary pathology, such as small vessel disease or epicardial vasospasm. Surgical therapy is only used as an adjunct to medical therapy and never as standalone approach due to lack of evidence. Potential surgical treatment options for INOCA are still rarely used compared to medical management. There is a lack of data on their benefits, and most of these treatments carry additional risks and complications associated with surgery, even when performed though minimally invasive approaches. There are no comparative studies to establish that one treatment may be better than another. Key studies of nonpharmacological treatments are summarized in Table 4. The strengths and limitations of the studies and their applicable groups are given in Table 5.

### 2.3. Transmyocardial Revascularization (TMR)

TMR is a surgical technique designed to improve myocardial perfusion, using a laser (usually a carbon dioxide (CO_2_) laser) to create small channels in the heart muscle, facilitating blood flow from the epicardial coronary vessels directly into the myocardium. TMR denervates the sympathetic nociceptors in the superficial myocardium and increases vascular endothelial growth factor (VEGF) release to induce angiogenesis [18,19]. Over time, the heart may develop collateral circulation, which can help relieve symptoms of angina and improve heart function.

TMR is typically performed with minimally invasive surgery, though it can also be performed with a traditional cardiac surgery approach. TMR has been primarily utilized for patients with advanced obstructive coronary artery disease, where traditional options like PCI or CABG were not possible. There is only limited research specifically investigating its efficacy in INOCA. In the subgroup of diabetics prone to microvascular, diffuse disease, CABG/TMR showed a greater relief of angina than CABG alone (93% vs. 63%, *p*  =  0.02) [20]. Previously, angina relief was attributed to placebo effects, as seen with sham thoracotomies and the Vineberg procedure. Most trials have lacked controls and have focused on refractory angina patients. More research is needed to determine whether TMR has beneficial short- and long-term effects in patients with microvascular dysfunction or endothelial dysfunction associated with INOCA. Improvements in perfusion may be temporary, and it is unclear whether it can reverse the microvascular abnormalities central to INOCA. Autologous bone marrow concentrate injections in conjunction with TMR channels into targeted ischemic tissue have been hypothesized to significantly enhance the angiogenic response compared with TMR alone.

Like any surgical procedure, TMR carries risks, and for INOCA patients those risks must be carefully considered and balanced with the uncertainty of the benefits of TMR. There are additional risks of wound infection, postoperative pain, perioperative myocardial infarction, and stroke, especially in the presence of ungraftable vessels. The initial myocardial inflammation also causes an initial decrease in myocardial function, which can increase mortality in severely impaired ventricles. In the meta-analysis by Brione et al., 30-day mortality as-treated was 6.8% in the TMLR group and 0.8% in the control group (pooled OR was 3.76, 95% CI 1.63 to 8.66), largely due to crossover from standard therapy [10].

### 2.4. Sympathectomy

The sympathetic nervous system (SNS) plays a crucial role in regulating vascular tone, and excessive activation or dysregulation of sympathetic pathways can contribute to the development of vasospasm [21,22].

Sympathetic denervation is a surgical procedure that targets the sympathetic pathway of the autonomic nervous system to treat specific cardiovascular diseases. Sympathetic denervation blocks the sympathetic nerves in the cervical ganglions innervating the heart, which can reduce coronary artery spasm and improve myocardial perfusion.

Sympathetic denervation can be achieved through a percutaneous sympathetic nerve ablation technique or surgical sympathectomy.

#### 2.4.1. Percutaneous Sympathetic Nerve Ablation

This minimally invasive procedure targets sympathetic nerve fibres near the coronary arteries using radiofrequency ablation or chemical neurolysis. The procedure is typically performed during coronary angiography, which allows the physician to localize the sympathetic nerve clusters and apply targeted energy or chemical agents to ablate the nerve fibres.

Radiofrequency ablation involves applying heat to selectively destroy the sympathetic nerve fibres, while chemical neurolysis uses agents such as phenol or ethanol to remove the nerves.

#### 2.4.2. Surgical Sympathectomy

Thoracic sympathectomy involves surgically cutting sympathetic nerve pathways in the chest. While this approach has been more commonly used in other areas (e.g., for hyperhidrosis or Raynaud’s disease), it has been proposed for refractory vasospastic angina, where vasospasm is a major contributor to ischemia. This procedure is more invasive and typically considered for patients with severe, intractable symptoms that do not respond to medical treatment or percutaneous interventions [23,24].

In some cases, an endoscopic approach may be used to perform sympathetic nerve blockade around the coronary arteries.

Sympathetic denervation can be considered as a treatment option in selected patients with INOCA. Although percutaneous approaches are minimally invasive, sympathetic denervation still carries procedural risks including wound infection, pneumothorax, chylothorax, chronic wound pain, and scarring. Removing or reducing sympathetic tone may result in an imbalance in autonomic regulation, potentially leading to unwanted side effects such as bradycardia, hypotension, or decreased contractility in some cases. The long-term effectiveness of sympathetic denervation in INOCA patients remains unclear. For these reasons, sympathetic denervation for INOCA is still considered experimental and is not yet widely adopted in clinical practice. More studies are needed to validate its efficacy and safety.

### 2.5. Coronary Sinus Reducer

The coronary sinus reducer is an interventional device that is being explored as a possible treatment option for patients with INOCA. The coronary sinus reducer is a stainless steel, hourglass-shaped endoluminal device, which is percutaneously implanted into the coronary sinus through an expandable balloon, to increase coronary venous pressure in order to mitigate coronary microvascular resistance. The concept behind this new therapy is that elevating pressure in the coronary venous system can cause dilatation of the subendocardial arterioles, resulting in a significant reduction in vascular resistance in this area and a possible redistribution of blood flow.

A double-blind sham-controlled trial (COSIRA trial) conducted in patients with refractory angina and obstructive coronary artery disease demonstrated that the implantation of the coronary sinus reducer alleviated refractory angina symptoms and improved quality of life [11]. These findings appear to be longstanding, as the multicenter observational REDUCER-I registry consistently reported sustained improvement in angina symptoms and quality of life at three years after implantation of coronary sinus reducer in participants from the COSIRA trial [17].

Recent studies have specifically evaluated the coronary sinus reducer implants as a possible treatment for INOCA patients. A case study reported a marked reduction in angina symptoms, as well as improved global myocardial perfusion and overall quality of life in one INOCA patient six months following coronary sinus reducer implantation [13]. Furthermore, a phase II trial published by Tryon D et al. reported significant improvement in coronary blood flow, coronary flow reserve, and angina symptoms in 30 patients with INOCA following coronary sinus reducer implantation [15]. Interestingly, the recent ORBITA-COSMIC trial assessed coronary sinus reducer implants for patients with stable coronary artery disease, ischemia, and no further options for antianginal therapy. This double-blind, placebo-controlled, multicenter study found no improvement in myocardial blood flow six months after implantation, but did report significantly reduced daily angina episodes, as reported via the designated smartphone ORBITA app [16].

Trials of coronary sinus reducer for refractory angina and INOCA, including the REDUCER-I multicenter “real-world” observational registry (refractory angina), have shown sustained improvements in angina class and survival over many years. However, registry and INOCA trial data lack a control arm. INOCA trial data, however, has objective evidence of improvement in physiological tests that are markers of severity.

The ESC (2023) guidelines for chronic angina refractory to other treatments provide a Class 2b recommendation (grade of evidence B) for use of the coronary sinus reducer in experienced centers [25]. There are several ongoing randomized control trials evaluating the use of coronary sinus reducer in ANOCA/INOCA, such as Coronary Sinus Reducer for the Treatment of Refractory Microvascular Angina (COSIMA; NCT04606459), and the Efficacy of the Coronary Sinus Reducer in Patients with Refractory Angina II (COSIRA-II; NCT05102019), that may change recommendations in the future.

#### 2.5.1. Autologous Stem Cell Therapy

Emerging therapies, such as the use of stem cells to treat coronary microvascular dysfunction, have been investigated as potential interventions for INOCA. The main goal is to use the patient’s own stem cells to promote the repair and regeneration of damaged blood vessels.

Corban et al. (2022) reported promising improvement in coronary flow reserve, angina symptoms, and quality of life with intracoronary infusion of autologous CD34+ cells in patients with INOCA [12]. Outcomes from the IMPROvE-CED trial also demonstrated safety and efficacy, with marked improvements in angina classification and sublingual GTN usage six months following a single infusion of CD34+ cells into the left anterior descending coronary artery of 20 INOCA patients, compared to 51 historic INOCA patients on maximal medical therapy [14].

Ongoing studies such as the ESCaPE-CMD Trial and FREEDOM Trial are currently investigating the therapeutic potential, efficacy, and safety of CD34+ cell therapy.

Most trials are still in early phases, and stem cell therapy remains experimental and is not yet used in clinical practice. It is still unclear whether stem cell therapy will provide long-term benefits for patients with microvascular dysfunction or endothelial dysfunction [3]. However, stem cell therapy may have potential as an adjunct to other treatments for maximum benefit.

#### 2.5.2. Selective Surgical Cardiac Vein Retroperfusion

Coronary artery bypass grafting (CABG) and percutaneous coronary intervention (PCI) are rarely indicated in the case of INOCA, as there is no significant epicardial coronary artery stenosis.

There have been reports and case series of surgical revascularization with arterial or venous conduits of the coronary veins rather than coronary arteries, to improve myocardial perfusion retrogradely [9]. These were largely related to non-graftable coronary arteries due to diffuse disease or small vessels and did not clearly investigate or identify INOCA as the primary pathophysiology. Initial results in experimental pig models suggested that surgical venous retroperfusion of the vena cordis magna with proximal ligation and global retroperfusion with ligation of the azygous vein could improve long-term survival after acute occlusion of the left anterior descending artery [26,27]. Another similar study in pigs showed that selective cardiac venous arterialization of the left anterior descending vein and its proximal ligation after anastomosing the left internal mammary artery could reduce the infarct size by more than 50%, while protecting cardiac performance [28].

## 3. Conclusions

INOCA is a complex condition that is poorly diagnosed and needs multidisciplinary management. Medical management remains the cornerstone of treatment. Selected surgical treatments are emerging as adjuncts for patients refractory to pharmacological therapy.

Among these, the coronary sinus reducer has shown improvement of symptoms and quality of life in promising early trials and may represent a minimally invasive strategy for enhancing microvascular perfusion. TMR offers potential symptomatic relief by promoting collateral circulation but currently lacks strong evidence for INOCA. Sympathetic denervation might be considered to relieve symptoms in patients with vasospastic angina but remains experimental with concerns about long-term efficacy. Targeted stem cell therapies are also emerging as potential therapies for this treatment-refractory cohort.

Most of these treatments remain experimental, and large randomized controlled trials are essential before routine clinical adoption.

## Figures and Tables

**Table 1:** Salient features of coronary artery disease of the macro- and microvasculature.

Macrovasculature (Epicardial CAD)		Microvasculature (INOCA)		DUAL Micro- and Macrovasculature	
Pathophysiology	Investigations	Pathophysiology	Investigations	Pathophysiology	Investigations
Stenotic	CTCA/CA shows discreet stenosis/diffuse disease FFR positive	Structural/functional -Cardiovascular disease -Endothelial dysfunction -Ventricular hypertrophy -Cardiomyopathies	Invasive physiologic assessment FFR > 0.80 or NHPR > 0.89 CFR < 2.0–2.5 IMR > 25 U or HMR >2.5 mm Hg/cm/s	Atherosclerotic disease Vasospastic disease	Positive CA/CTCA with positive microvascular physiological assessment
Ectatic	CTCA/CA	Microvascular vasospastic	Ischemia on vasoreactive testing No epicardial artery constriction Chest pain Ischemic ECG changes (ST-segment depression or elevation >0.1 mV) in at least two contiguous leads		
Myocardial bridging/anomalous course	CTCA shows external compression CMR/stress echo shows Ischemic positive	Diffuse mixed/isolated microvascular -Cardiovascular disease -Atherosclerosis	FFR < 0.80 or NHPR < 0.89 with gradual step-up on pull-back Intravascular imaging may show diffuse disease		
Epicardial vasospastic	Vasoreactive test positive				

CAD—Coronary artery disease, INOCA—ischemia with no obstructive coronary artery disease, CTCA—CT coronary angiography, CA—coronary angiogram, FFR—fractional flow reserve, NHPR—non-hyperemic pressure ratio, IMR—intramyocardial resistance, and HMR—hyperemic microvascular resistance.

**Table 2:** Diagnostic tests for INOCA.

Imaging Tests		Physiological Tests	
Coronary Angiography (CA)	Gold standard for epicardial disease resolution—0.1–0.2 mm cannot image microvasculature	Fractional Flow Reserve (FFR)	Invasive. Measures pressure drops across the circulation. Typically values less than 0.80 are considered significant (indexed FFR < 0.89).
CT Coronary Angiogram (CTCA)	Less invasive than CA—lower resolution of 0.3–0.4 mm	Coronary Flow Reserve (CFR)	Microvascular dysfunction, key marker of INOCA. <2.0 is impaired reserve. Correlates with clinical outcomes.
Cardiac Magnetic Resonance Imaging (cMRI)	Myocardial perfusion and ischemic areas Late Gadolinium Enhancement (LGE) identify myocardial injury or fibrosis	Non-Hyperemic Pressure Ratio (NHPR)	Indicative of coronary microvascular resistance. NHPR < 0.89 with gradual step-up on pull-back.
Positron Emission Tomography (PET)	Myocardial perfusion, coronary blood flow, and metabolic activity highly sensitive in detecting subclinical myocardial ischemia	Hyperemic Microvascular Resistance (HMR)	Indicative of microvascular dysfunction HMR > 2.5 mm Hg/cm/s is high.
Intravascular Ultrasound (IVUS)	Cross-sectional plaque burden and signs of microvascular disease in surrounding tissue.	Index of Microcirculatory Resistance (IMR)	IMR > 25 U is high.
Optical Coherence Tomography (OCT)	Endothelial and microvascular abnormalities with infra-red light	Stress Testing— Exercise Treadmill Testing and Dobutamine Stress Testing (Stress Echocardiography)	Assesses silent myocardial ischemia.
		Nuclear Stress Tests (SPECT/PET)	Show regional myocardial perfusion defects.

**Table 3:** Pharmacological and medical therapy of INOCA.

Anti-Anginal Agents		Lifestyle Modification
Nitrates	Improve coronary arteries dilatation and myocardial perfusion. Relieve symptoms of chest pain or discomfort due to reduced coronary blood flow Do not treat underlying microvascular dysfunction in INOCA.	Exercise and Physical Activity
Beta-Blockers	Reduce heart rate, myocardial contractility and myocardial oxygen demand. Improve endothelial function by reducing sympathetic stimulation. Decreasing oxidative stress in the coronary microcirculation.	
Calcium Channel Blockers	Reduce vascular resistance by coronary dilatation. Reduce myocardial oxygen demand by lowering heart rate and contractility. Effective in treating both microvascular dysfunction and vasospasm.	
Ranolazine	Reduces intracellular calcium overload, improves myocardial relaxation and perfusion. Active in refractory angina and non-responders to other anti-anginals. Improves microvascular dysfunction without affecting heart rate or blood pressure_._	
Nicorandil	Dual properties of nitrates at low doses (epicardial vessels) and ATP-sensitive K^+^ channel opener at high doses (decreases microvascular resistance). Effective even where nitrates are not effective.	
Antiplatelet therapy Aspirin	reduces inflammation and risk of thrombotic events stabilizes non-obstructive atherosclerotic plaques by platelet inhibition.	Weight Management and Healthy Diet
Clopidogrel (P2Y12 inhibitors)	reduce increased thrombotic risk imrove thrombotic microvascular injury.	
Statins	lower cholesterol levels by HMGCoA reductase inhibition. improve endothelial function and reduce inflammation. reduce the overall risk of cardiovascular events.	Stress Management and Mental Health
ACE Inhibitors and ARBs	lower blood pressure, reduce strain by inhibiting renin-angiotensin-aldosterone system. promote vasodilation and improve endothelial function.	Smoking Cessation and Alcohol Moderation
If current inhibitors	New class of drugs; blocks If pacemaker current (funny current) in the sinus node (SA) reduces heart rate without affecting cardiac contractility by blocking the HCN channels and delaying the repolarization. Mainly indicated for BB/CCB intolerance as acts through SA node	
Antiinflammatory drugs and steroids	Reduce inflammation and tissue odema and improve microcirculation in inflammatory cardiomyopathies	

**Table 4:** Nonpharmacological studies of refractory angina/INOCA.

Study Name Author Year (PMID) [Ref]	Design	Population	No. Patients	Intervention	Primary Outcomes	Results
Sadaba et al. 2004 (15464519) [9]	Case report	Refractory angina	1	CABG	Angina	Complete relief of angina.
Briones et al. 2015 (25721946) [10]	Meta-analysis (seven studies)	Refractory angina—not for PCI/CABG	1137	TMR vs. OMT	Mortality angina score	The 30-day mortality was at 6.8% following TMLR compared with 0.8% OMT, and subjective improvements in angina score were compounded by bias.
CORSIRA Verheye et al. 2015 (25651246) [11]	Randomized control trial	Refractory angina	104 52 CSR 52 Sham	CSR vs. Sham	+2 CCS angina class improvement	Primary endpoint was achieved in 35% (18/52) of the reducer-treated patients versus 15% (8/52) of the sham (*p* = 0.02). At least one CCS angina class improvement in 71% vs. 42% in the sham (*p* = 0.003).QoL improved significantly in the reducer group compared to the sham (17.6 vs. 7.6 points, *p* = 0.03).
IMPROvE-CED Corban et al. 2022 (34923853) [12]	Clinical trial	INOCA	20	Intracoronary (LAD) autologous CD34+ cell therapy	Safety, angina score, sublingual GTN usage, and exercise tolerance	The infusion was safe and resulted in statistically significant decrease in Canadian Cardiovascular Society angina class (*p* = 0.00018) and sublingual GTN use (*p* = 0.00047), but no significant improvement in exercise tolerance compared with historical control patients, following a one-off infusion via the LAD
Cheng et al. 2022 (36415685) [13]	Case report	INOCA	1	CSR	Symptom relief, MPR, and ischemia burden at six months	A marked reduction in ischemia burden, improved global MPR, symptoms, and quality of life
NCT03508609 Henry et al. 2022 (35067072) [14]	Clinical trial	INOCA	20	Intracoronary (LAD) autologous CD34+ cell therapy	Efficacy: coronary flow reserve, angina score, and exercise tolerance	Improved coronary flow reserve (CRF) at six months (*p* < 0.005); decreased angina (*p* < 0004); improved angina class (*p* < 0.001); and improved quality of life score *p* < 0.03). No serious adverse events noted. Comparisons made with patient scores at baseline (pre-intervention).
G200153 Tyron et al. 2024 (39520443) [15]	Clinical trial	INOCA	30	CSR	Coronary flow reserve (CFR); coronary blood flow (CBF)	Increased CFR from 2.1 to 2.7 (*p* = 0.0011); increased CBF from 11% to 11.5% (*p* = 0.042); improved CCS angina class from 4 to 2 (*p* < 0.0001); and improved quality of life across all questionnaire domains (*p* < 0.0006).
ORBITA-COSMIC Foley et al. 2024 (38604209) [16]	Randomized control trial	Refractory angina	51 25—CSR 26—placebo	CSR vs. placebo	Angina improvement at 6 m (ORBITA app)	Myocardial blood flow (MBF) in ischaemic segments did not improve with CSR compared with placebo (difference 0·06 mL/min per g [95% CI −0.09 to 0.20]; Pr(Benefit) = 78.8%). Daily angina episodes reduced with CSR compared with placebo (OR 1.40 [95% CI 1.08 to 1.83]; Pr(Benefit) = 99.4%).
REDUCER-I Verheye et al. 2024 (39520437) [17]	Real world multicenter registry	Refractory angina	400	CSR	Improved CCS score at six months and improved Seattle Angina Questionnaire score at six months	69.8% patients improved by >1 CCS class at six months and interim three year results showed sustained improvements (*p* < 0.0001).

CABG—coronary artery bypass grafting, PCI—percutaneous coronary intervention, CSR—coronary sinus reducer, CI— confidence interval, and OMT—optimal medical therapy.

**Table 5:** Strengths and limitations of the included studies and applicable groups.

Study Name, Author Year (PMID) [Ref]	Evidence Level	Population	Strengths	Limitations	Pathophysiological Groups Likely to Benefit
Sadaba et al. 2004 (15464519) [9]	Case report	Refractory angina	-	Limited evidence Pathophysiology is unclear Subjective endpoint	Unclear
Briones et al. 2015 (25721946) [10]	Meta-analysis (seven studies)	Refractory angina—not for PCI/CABG	Large evidence base Meta-analysis of seven studies Control arm	Mainly observational studies Cohorts not INOCA Pathophysiology unclear No physiological evaluation for pathophysiology No post intervention physiological tests Subjective endpoints Placebo effects	Most with refractory angina with multiple pathophysiologies
CORSIRA Verheye et al. 2015 (25651246) [11]	Randomized control trial	Refractory angina	Control group Randomized groups First RCT for CSR	Small numbers Not INOCA Pathophysiology unclear Not generalizable No physiological measures Subjective endpoints	Diffuse coronary disease Ungraftable vessels Microvascular disease Failed OMT
IMPROvE-CED Corban et al. 2022 (34923853) [12]	Clinical trial	INOCA	Clinical trail setting Well-defined INOCA group Clear methodology and endpoints	Limited numbers Very controlled group Confounders with other treatments No imaging Subjective endpoints	Diffuse coronary disease Ungraftable vessels Microvascular disease Failed OMT
Cheng et al. 2022 (36415685) [13]	Case report	INOCA	-	Limited report Subjective assessment Not generalizable	-
NCT03508609 Henry et al. 2022 (35067072) [14]	Clinical trial	INOCA	Clinical trail setting Well-defined INOCA group Clear methodology and endpoints CRF measured	Limited numbers Very controlled group Confounders with other treatments Subjective endpoints Results not generalizable	Microvascular disease Failed OMT
G200153 Tyron et al. 2024 (39520443) [15]	Clinical trial	INOCA	Clinical trail setting Well-defined group Clear methodology and endpoints CFR, CBR measured	Limited numbers Very controlled group Confounders with other treatments No imaging Subjective endpoints Results not generalizable	Microvascular disease Failed OMT
ORBITA-COSMIC Foley et al. 2024 (38604209) [16]	Randomized control trial	Refractory angina	Clinical trail setting Well-defined group Clear methodology and endpoints MBF measurements	Limited numbers Very controlled group Confounders with other treatments No imaging No physiological assessment Subjective endpoints Results may be generalizable	Microvascular disease Failed OMT
REDUCER-I Verheye et al. 2024 (39520437) [17]	Real world multicenter registry	Refractory angina	Real world setting Large number Long term follow-up	Limited numbers Ill defined group Confounders with other treatments No imaging No physiological assessment Subjective endpoints Results may be generalizable	Microvascular disease Failed OMT

CABG—coronary artery bypass grafting, PCI—percutaneous coronary intervention, CSR—coronary sinus reducer, CI— confidence interval, and OMT—optimal medical therapy.

## Data Availability

Data available on request. The data underlying this article will be shared on reasonable request to the corresponding author.
